# Contextualising the job demands–resources model: a cross-sectional study of the psychosocial work environment across different healthcare professions

**DOI:** 10.1186/s12960-024-00958-1

**Published:** 2024-11-19

**Authors:** Britta Elsert Gynning, Elin Karlsson, Kevin Teoh, Per Gustavsson, Filip Christiansen, Emma Brulin

**Affiliations:** 1 The Department of Occupational Medicine, Institute of Environmental Medicine, Karolinska Institutet, Nobels Väg 13, 171 65 Solna, Stockholm Sweden; 2 The Department of Society and Health, Institute of Health, Medicine and Care, Linköping University, Linköping, Sweden; 3 The Department of Organizational Psychology, Clore Management Centre, Birkbeck University of London, London, UK; 4 Center for Occupational and Environmental Medicine, Region Stockholm, Sweden

**Keywords:** Job demands–resources model, Job demands, Job resources, Health care workers, Cross-sectional

## Abstract

**Background:**

The deteriorating psychosocial work environment among healthcare workers in Sweden, influenced by demanding working conditions and resource constraints, affects individual well-being and patient care quality. Healthcare workers, including physicians, registered nurses, and nursing assistants, often work interdependently and share workplaces, yet are three completely different professions. Nonetheless, comprehensive studies comparing their psychosocial work environments are scarce; often focusing on healthcare workers either separately or as a homogenous group, but rarely comparative.

**Aim:**

Utilising the job demands–resources model, this study investigated variations in the psychosocial work environment among Swedish healthcare workers. We wanted to identify how the antecedents of individual well-being, in the form of demands and resources, differed between healthcare workers.

**Method:**

Data from the 2022 Longitudinal Occupational Health Survey for Health Care in Sweden were analysed; the participants included 7589 physicians, registered nurses, and nursing assistants. The analysis involved descriptive statistics, including measures of means and analysis of covariance (ANCOVA), employing the Bonferroni correction for multiple post hoc comparisons. The ANCOVA was also stratified by working factors, including years of work experience and employment within the private/public sector.

**Results:**

The study revealed significant variations in how healthcare workers perceive their psychosocial work environment. Physicians faced the highest level of Quantitative Demands (mean (x̄) 3.15; 95% CI 3.11–3.19), while registered nurses reported the most Emotional Demands (x̄ 3.37; 95% CI 3.32–3.41). Nursing assistants had the highest grand means for the imbalance between Efforts and Rewards (Effort Reward Imbalance) (x̄ 1.49; 95% CI 1.49–1.49) and an imbalance between Work and Private Life (Work–Life Interference) (x̄ 3.20, 95% CI 3.15–3.25), along with limited resources. The stratified analysis showed that years of experience and the sector affected healthcare workers' perceptions of their psychosocial working environment. For example, registered nurses working in the private sector reported better working conditions than registered nurses working in the public sector. The situation for nursing assistants was reversed.

**Conclusion:**

Psychosocial work environments are experienced differently between and within healthcare professions in Sweden. This study provides crucial insights for improving workplace conditions and consequently enhancing healthcare professionals’ well-being and quality of patient care.

**Supplementary Information:**

The online version contains supplementary material available at 10.1186/s12960-024-00958-1.

## Background

Healthcare workers worldwide face escalating strains characterised by heavy workloads and increased absenteeism due to demanding working conditions and constrained resources [1–5]. While extensive research has examined the psychosocial work environment of healthcare workers, most studies often focus on specific professions or study them as a uniform group. Despite the diverse roles and responsibilities within healthcare, the interdependence of different healthcare professions necessitates a more comprehensive approach, simultaneously studying healthcare workers jointly, comparatively and independently, to, in the long run, unveil how the intricate dynamics of healthcare may implicate care and safety for patients [6–9].

The challenging psychosocial work environment faced by healthcare workers, marked by heightened job demands, role conflicts, an imbalance between effort and reward, and the imposition of illegitimate tasks, has consistently been associated with increased stress levels [3, 5, 9–15]. This stress, in turn, has the potential to result in adverse outcomes such as sickness absence and loss of competence, including lower quality of care [16–19]. Conversely, the availability of various job resources, including social support and a sense of control, has been demonstrated to enhance work engagement and reduce the likelihood of healthcare workers leaving their profession [4, 9, 20].

Previous research underscores how variation in healthcare workers’ psychosocial work environment^1^ may vary based on professional and sociocultural differences both between and within professions [10, 22–24]. A scoping review by McVicar [10] emphasised the need to consider interpersonal and inter-professional factors when researching differences in healthcare workers’ work environments to decipher how each specific context affects their well-being [10]. While previous Swedish studies have compared the work environment of healthcare professions [25–28] and shown differences between two professions, e.g., registered nurses versus nursing assistants [26] or physicians versus registered nurses [27], many often focused on one main outcome, such as job satisfaction [27], or one specific context, such as primary care [6]. Few previous studies have explicitly charted and compared the psychosocial work environment of Sweden’s three major healthcare professions: physicians, registered nurses, and nursing assistants. These comparison are crucial for understanding the similarities and differences within and between professions as well as the psychosocial work environment.

This study adapts the job demands–resources (JD-R) model developed by Demerouti et al. [29] to address the lack of comparative research across healthcare workers in Sweden. The JD-R model posits that each work setting harbours unique factors influencing job performance by impacting employee well-being. These factors are categorised as job demands or job resources, ultimately leading to burnout or work engagement [30, 31]. Job demands necessitate effort and deplete energy, incurring physiological and psychological costs [29]. Conversely, job resources increase motivation and have been hypothesised to buffer the effect of demands on health outcomes [29, 30]. However, what might be a demand or resource within one context or one group might not apply to others [9, 32, 33]. Therefore, while there have been studies concerning healthcare workers using the JD-R model (e.g., [3, 5, 10, 11, 13, 32, 33]), we cannot presume that the demands or resources (or the degree of them) will be the same for each of the three groups of healthcare workers within the Swedish context.

In this study, we draw on the JD-R model to investigate variations in the psychosocial work environment among Swedish healthcare workers, including physicians, registered nurses, and nursing assistants. Through this comparative analysis, we seek to unpack variations between healthcare professions in the experience of different job demands and job resources, paving the way for profession-specific interventions to enhance the individual work environment of healthcare workers within a uniform healthcare system.

## Method

### Study setting

This study is situated in Sweden, where hospital and primary healthcare are organised under 21 self-governing counties. Swedish healthcare is by large publicly funded by taxes with universal access. However, in recent decades, an increasing number of healthcare facilities (mostly in primary care) have become private, which can either (1) have public funding in order to keep universal access but are privately run or (2) be fully private, “fee-for-service” facilities. In this article, we will refer to the private sector including both the publicly funded private facilities and the fully private, while the public sector includes the fully publicly owned facilities. This evolution of the Swedish healthcare system dividing into public and private care has been propelled by increased patient numbers and reduced accessibility, thus private facilities were introduced to minimise the pressure on the public sector [34]. This has then prompted healthcare workers to choose between working within either a public or private employer sector. Each employer has its own composition of patients, demands and resources affecting the health care workers working environment [12, 35, 36]: e.g., public facilities often have both older and sicker patients compared to private facilities. Private facilities on the other hand are often more aware of financial incentives leading to a perception of an increased production of shorter visits and even skimming of patients [37]. Social care, including home care, is organised under the government of a self-governing municipality.

In Sweden, physicians and registered nurses must have formal medical or nursing training from the university while the formal training for nursing assistants may differ but do not include university-level studies. Registered nurses receive a certificate immediately after 3 years of schooling and may start working in healthcare directly. Physicians need 1.5 years of additional supervised clinical rotations before they can have their certificate—although this is now changing. After certification, physicians start their residency training of a minimum of seven years. Work experience, aligning with seniority, has been noted to influence the perception of one’s work environment [1, 10, 38], within the Swedish healthcare system, especially for physicians and registered nurses, less working experience has meant a higher amount of job demands and fewer accessible job resources [39].

### Study design, study participation and data collection

This study applied data from the Longitudinal Occupational Health Survey for HealthCare in Sweden (LOHHCS). The LOHHCS cohort included a sample of practising physicians, registered nurses, and nursing assistants in Sweden. For more information, see Hagqvist et al. [40].

The survey sample of physicians included 7908 individuals, 2712 of whom answered the survey (34.3%). The sample of registered nurses included 7790 individuals, 2903 of whom responded (33.5%). For nursing assistants, the sample included 7748 individuals, of whom 2043 responded (26.4%). The final study population included 7658 individuals; after excluding individuals over the age of 69, the final analytical sample consisted of 7589 individuals, 2643 physicians (34.8%), 2903 registered nurses (38.3%) and 2043 nursing assistants (26.9%).

The data for all healthcare workers were collected in 2022. This study was approved by the Swedish Ethical Review Authority (2021-05574-02; 2022-00310-02; 2022-02765-02).

### Study measures

To investigate variations in the psychosocial work environment among Swedish healthcare workers, the JD-R model was utilised as the overarching theoretical framework. This was operationalised by a combination of different study measures (based on both existing theories and models) implemented as either job demands and job resources. Each of the job demands and job resources was selected based on a comprehensive review authored by the Swedish Agency for Work Environment Expertise, which concluded specific job demands (Quantitative- and Emotional demands, Effort Reward Imbalance, Illegitimate Tasks and Work–Life Interference) and job resources (Control and Social Support) as central for the work environment of Swedish healthcare workers [39]. Cronbach’s alpha (α) for each measurement of job demand and job resource for the total sample is presented in the methods section. Items and response alternatives for each study measure and Cronbach’s alpha for each healthcare profession can be found in the supplementary material (Supplementary Table A). For the analysis all study measures were oriented so that higher values indicate more demands or more resources; scales not originally aligned were reverse-coded.

#### Demands

Q*uantitative-* and *Emotional Demands* were measured by three and one item, respectively, retrieved from the validated survey Copenhagen Psychosocial Questionnaire (COPSOQIII) developed for the examination of the organisational and social working environment [41]. Respondents rated both types of demands on a 5-point Likert scale ranging from (1) “Always” to (5) “Never/Almost never”. The three items of Quantitative Demands were compiled into a grand mean score ranging from 1 to 5 (α: 0.866).

*Effort Reward Imbalance (ERI)* examines the balance between an individual’s work effort and subsequent rewards [42]. The ERI is measured by a ratio formula,^2^ accounting for the unequal number of items in both Effort (3 items; α: 0.782) and Reward (7 items; α:0.769). Each item was measured using a 4-point Likert scale (Ranging from “Strongly disagree” to “Strongly agree”) utilising the validated ERI scale [42]. The ERI-ratio cut-off value was set at 1: < 1 indicates more rewards than effort, = 1 signifies balance, and > 1 indicates an imbalance, where more effort than rewards has been associated with increased stress and deteriorated health [42].

*Illegitimate Work Tasks (IWT)* were assessed through the Bern Illegitimate Task Scale (BITS) [43] and included two main categories: unnecessary tasks (avoidable or pointless tasks) and unreasonable tasks (tasks outside of the professional role that should be handled by others). Both categories were evaluated using four items each, employing a 5-point Likert scale ranging from (1) “Very often” to (5) “Never”. The two categories were generated using a grand mean score ranging from 1 to 5 (α: 0.770 and 0.839, respectively).

*Work to Life Interference (WLI),* pertains to an imbalance between coping with expectations and time management from one’s work role in parallel with expectations from one’s private life role [44]. The WLI was assessed using the validated scale from Fisher et al. [45] and refers to an inter-role conflict between balancing one’s work role with one’s life role (family, friends, leisure), creating strain and stress [44, 45]. The WLI contains five items on a 5-point Likert scale ranging from (1) “Not at all” to (5) “Almost all the time”. The five items were compiled into an index using a grand mean score ranging from 1 to 5 (α: 0.927).

#### Resources

*Control* was assessed through three categories: (1) *Work Content Control,* (2) *Work Time Control* and (3) *Influence* (the possibility of making clinical decisions and giving high-quality care). Each item was measured on a 6-point Likert scale ranging from (1) “To a very high degree”, (5) “To a very low degree”, and (6) “Not relevant”. Work Content Control (5 items), Work Time Control (3 items) and Influence (3 items) were indexed through factor analysis,^3^ which indicated the best fit for each item (α: 0.908; 0.911 & 0.777, respectively). For each control index, the option “Not relevant” was excluded and handled as missing. Questions for Work Content Control and Work Time Control were drawn from the Swedish Longitudinal Occupational Survey of Health (SLOSH) [46, 47]. The questions for Influence included items such as “In my workplace, I have the freedom to make clinical decisions that meet the needs of the patient”.

*Social support* was assessed using two categories from the validated survey COPSOQIII [41]: (1) Manegerial Support and (2) Collegial Support. Each category featured one item on a 5-point Likert scale, from (1) “Always” to (5) “Never/almost never”, with the fifth option being “Not relevant”.

#### Confounders and stratifying variables

The analysis considered several potentially confounding variables that were chosen on recommendations by Becker et al. [48] and notions of influence on how individuals within the healthcare sector perceive their work environment. The confounding demographic variables included *age* [10, 49]*, sex* [10, 28, 50]*,* and *birth country* [10, 51, 52]; the profession-specific variables included *working hours* [22, 49] and years of *working experience* [1, 10]*.*

The demographic variables (age, sex, and birth country), along with the healthcare workers’ *place of work* and their *county of work,* were also used to regulate skewed sampling from collection to avoid selection bias.

Concerning stratification, years of *working experience* and employment within the *private* vs*. public sector* were used as stratification variables to uncover differences within each profession*.*

In the analysis, *age* was treated as a continuous variable, measured in years but categorised into quartiles for demographic description (i.e. < 36, 37–47, 48–57, > 58). *Sex* was categorised as male or female. *Birth country* was dichotomised into born within Sweden and born outside of Sweden.

*Workplace* was categorised into three main workplaces for healthcare workers in Sweden (primary care, municipality, and hospital), along with a fourth category, “Other”, which included work within occupational health service, consulting or other. *The county of work* represents the 21 self-governing counties of Sweden.

*Working hours* were categorised as < 36 h/week, between 36 and 40 h and > 40 h/week.

*Working experience,* both a confounder and stratification variable*,* was categorised into < 5 years of working experience, 5–15 years and > 15 years.

*Private* vs*. public sector* employment was measured by dichotomising the variable of main employment into working for either a private or public employer, excluding the options of working for governments, staffing agencies or self-employment.

### Statistical analysis

All analyses were performed using SPSS version 28.0.

The analysis involved the calculation of descriptive summary statistics, encompassing demographic and work characteristics, as well as the means of each demand and resource across each profession. Additionally, to identify significant inter-professional differences between the three professions’ psychosocial work environments, an analysis of covariance (ANCOVA) was utilised employing the Bonferroni correction for multiple post hoc comparisons at the 0.05 significance-level. To uncover interpersonal differences within each profession, the ANCOVA was stratified by work characteristics, including years of work experience and employment sector.

## Results

In the following section, we present the findings of the current study. We begin with an overview of the demographic and work characteristics of individuals in each profession. We subsequently compare grand means across profession, which are thereafter stratified by two separate work characteristics.

### Study sample

Table 1 describes the sample characteristics. The majority of the healthcare workers in our study were women (79.4%), and this trend continued across all three professions, with physicians being the most evenly distributed in terms of sex (52.9%). Ages ranged from 21 to 69 years, although the lowest age among physicians was 25. The physicians had a mean age of 44 years, the registered nurses had a mean age of 47 years, and the nursing assistants had an age of 51 years.

**Table 1:** Demographic- and work characteristics by each healthcare profession

	Total	Physicians	Registered nurses	Nurse assistants
	*n* (%)	*n* (%)	*n* (%)	*n* (%)
Total	7589 (100%)	2643 (34.8%)	2903 (38.3%)	2043 (26.9%)
Demographics				
Sex				
Men	1563 (20.6%)	1114 (42.1%)	288 (9.9%)	161 (7.9%)
Women	6026 (79.4%)	1529 (57.9%)	2615 (90.1%)	1882 (92.1%)
Age				
< 36 years	1840 (24.2%)	811 (30.7%)	741 (25.5%)	288 (14.1%)
37–47 years	1941 (25.6%)	883 (33.4%)	724 (24.9%)	334 (16.3%)
48–57 years	1861 (24.5%)	491 (18.6%)	700 (24.1%)	670 (32.8%)
> 58 years	1947 (25.7%)	458 (17.3%)	738 (25.4%)	751 (36.8%)
Birth country				
Within Sweden	6438 (85.4%)	2117 (80.8%)	2641 (91.7%)	1680 (82.2%)
Outside Sweden	1105 (14.6%)	502 (19.2%)	240 (8.3%)	363 (17.8%)
Work factors				
Years of working experience				
< 5 years	1159 (15.3%)	547 (20.7%)	474 (16.4%)	138 (6.8%)
5–15 years	2515 (33.3%)	1044 (39.6%)	869 (30.0%)	602 (29.6%)
> 15 years	3889 (51.4%)	1046 (39.7%)	1552 (53.6%)	1291 (63.6%)
Self-estimated number of working hours per week				
< 36 h	2073 (27.5%)	369 (14.0%)	883 (30.6%)	821 (40.7%)
36–40 h	2451 (32.5%)	501 (19.0%)	1108 (38.4%)	842 (41.8%)
> 40 h	3011 (40.0%)	1761 (66.9%)	898 (31.1%)	352 (17.5%)
Public/private sector				
Public	6436 (88.1%)	2174 (85.3%)	2456 (88.6%)	1806 (90.8%)
Private	872 (11.9%)	375 (14.7%)	315 (11.4%)	872 (11.9%)

There was a large variation between professions regarding working hours. Physicians reported working more than the Swedish standard of 40 h per week (66.9%), while registered nurses and nursing assistants reported working between 36 and 40 h per week most often (38.4% and 41.8%, respectively). Most of the participants worked publicly (88.1%).

### Grand means per profession

In Table 2, the grand means for each variable and profession are described, along with p-values (*p* > 0.05) for differences. Adjusting for potential confounders, physicians faced the highest level of Quantitative Demands (mean (x̄) 3.15), while registered nurses reported most Emotional Demands (x̄ 3.37), although these demands were significantly different only from those of nursing assistants. Nursing assistants had the highest grand means for ERI (x̄ 1.49) and WLI (x̄ 3.20) after adjustment.

**Table 2:** Mean values for job demands and job resources divided by healthcare profession adjusted by confounders†

	Total		Physicians		Registered nurses		Nursing assistants		*P*-values		
	Mean (SD)	*n*	Mean (95% CI)	*n*	Mean (95% CI)	*n*	Mean (95% CI)	*n*	*P*-value between physicians and registered nurses	*P*-value between physicians and nursing assistants	*P*-value between registered nurses and nursing assistants
Job Demands											
Quantitative demands^1^	2.92 (0.94)	7370	3.15 (3.11–3.19)	2576	2.86 (2.83–2.89)	2830	2.70 (2.66–2.74)	1964	0.001*	0.001*	0.001*
Emotional demands^1^	2.30 (1.16)	7392	3.30 (3.25–3.35)	2580	3.37 (3.32–3.41)	2840	3.21 (3.15–3.26)	1972	0.147	0.058	0.001*
Effort Reward Imbalance^2^	1.35 (0.57)	7170	1.19 (1.16–1.23)	2508	1.38 (1.35–1.41)	2776	1.51 (1.47–1.54)	1886	0.001*	0.001*	0.001*
Illegitimate tasks—unneccessary^1^	3.05 (0.81)	7326	3.08 (3.05–3.12)	2569	3.01 (2.98–3.04)	2813	3.04 (3.01–3.08)	1944	0.010*	0.447	0.528
Illegitimate tasks—unreasonable^1^	2.96 (0.85)	7372	2.93 (2.90–2.97)	2580	2.93 (2.90–2.97)	2827	3.03 (2.99–3.07)	1965	1.00	0.002*	1.00
Work–Life Interference^1^	3.11 (1.06)	7348	3.10 (3.05–3.14)	2572	3.05 (3.01–3.09)	2825	3.20 (3.15–3.25)	1951	0.482	0.009*	0.001*
Job Resources											
Work content control^1^	2.55 (1.16)	4914	2.35 (2.30–2.40)	2180	2.93 (2.88–2.99)	1856	2.22 (2.14–2.29)	878	0.001*	0.015*	0.001*
Work time control^1^	2.29 (1.25)	6466	2.48 (2.43–2.54)	2403	2.35 (2.30–2.39)	2530	1.91 (1.85–1.97)	1533	0.001*	0.001*	0.001*
Influence^1^	3.43 (.83)	6867	3.64 (3.61–3.67)	2476	3.52 (3.49–3.55)	2714	2.97 (2.93–3.01)	1677	0.001*	0.001*	0.001*
Social support—managers^1^	3.58 (1.18)	7114	3.65 (3.59–3.70)	2454	3.57 (3.53–3.62)	2742	3.50 (3.44–3.56)	1918	0.134	0.001*	0.130
Social support—colleagues^1^	4.35 (0.78)	7355	4.33 (4.29–4.36)	2565	4.42 (4.39–4.45)	2818	4.28 (4.25–4.32)	1972	0.001*	0.346	0.001*

^*^p < 0.05

^†^Confounders include sex, birth country, working hours, working experience, place of work and county of work

Note that several study measurement scales were reverse-coded to ensure that higher scores uniformly indicate greater demands or greater resources. The original coding of all scales is documented in the supplementary material

Scales ranges in the table:

^1^Scale 1–5

^2^Scale 0–4

The differences in ERI between professions increased after we adjusted for confounders, suggesting that the variations in ERI are more likely attributed to the professions themselves rather than to external factors. Notably, all professions reported an ERI mean over 1, indicating a shared experience of exerting more effort than receiving a reward.

Regarding available resources (Table 2), after adjusting for potential confounders, the nursing assistants reported the lowest grand means across all the researched resources. The most notable difference was in terms of experience of Influence where nursing assistants reported a mean of 2.97 points, whereas physicians reported a mean of 3.64 points and registered nurses 3.52 points.

#### Stratified analysis by years of working experience and private vs. public sector

When stratified by years of working experience and adjusted for potential confounders (Table 3), physicians with > 15 years of working experience reported the highest grand mean for Quantitative Demands (x̄ 3.37). Concerning resources, however, physicians with < 5 years of working experience reported lower Work Time Control (x̄ 2.19) and Influence (x̄ 3.50) than did more experienced colleagues.

**Table 3:** Mean values for job demands and job resources stratified by years of working experience for each profession adjusted by confounders†

		Years of working experience						Bonferroni *p*-value		
		< 5 years		5–15 years		> 15 years				
		Mean (95% CI)	*n*	Mean (95% CI)	n	Mean (95% CI)	*n*	< 5 years of work experience vs. 5–15 years of work experience	< 5 years of work experience vs. > 15 years of work experience	5–15 years of work experience vs. > 15 years of working experience
Job Demands										
Quantitative demands^1^	Physicians	2.98 (2.88–3.08)	540	3.22 (3.16–3.28)	1024	3.37 (3.29–3.45)	1012	0.001*	0.001*	0.039*
	Registered nurses	2.99 (2.89–3.09)	466	2.81 (2.75–2.88)	852	2.77 (2.72–2.83)	1512	0.001*	0.003*	1.00
	Nursing assistants	2.66 (2.50–2.83)	129	2.70 (2.62–2.77)	583	2.62 (2.57–2.68)	1252	1.00	1.00	0.444
Emotional demands^1^	Physicians	3.14 (3.01–3.27)	539	3.36 (3.28–3.44)	1025	3.37 (3.26–3.47)	1016	0.003*	0.070	1.00
	Registered nurses	3.42 (3.29–3.55)	467	3.42 (3.33–3.50)	853	3.34 (3.27–3.41)	1520	1.00	1.00	0.662
	Nursing assistants	2.93 (2.72–3.15)	129	3.20 (3.10–3.30)	584	3.18 (3.12–3.25)	1259	0.056	0.107	1.00
Effort Reward Imbalance^2^	Physicians	1.20 (1.15–1.26)	522	1.26 (1.23–1.30)	1002	1.24 (1.20–1.29)	984	0.124	1.00	1.00
	Registered nurses	1.49 (1.42–1.55)	456	1.35 (1.31–1.39)	844	1.34 (1.30–1.38)	1476	0.001*	0.002*	1.00
	Nursing assistants	1.40 (1.28–1.52)	124	1.51 (1.46–1.57)	553	1.44 (1.40–1.48)	1209	0.193	1.00	0.132
Illegitimate tasks—unneccessary^1^	Physicians	3.09 (3.01–3.18)	538	3.23 (3.18–3.28)	1018	3.20 (3.13–3.27)	1013	0.005*	0.316	1.00
	Registered nurses	3.11 (3.01–3.20)	465	3.00 (2.93–3.06)	849	2.92 (2.87–2.97)	1499	0.063	0.008*	0.305
	Nursing assistants	2.82 (2.67–2.98)	127	3.03 (2.96–3.10)	579	2.94 (2.89–2.99)	1238	0.033*	0.502	0.205
Illegitimate tasks—unreasonable^1^	Physicians	3.01 (2.93–3.10)	538	3.03 (2.98–3.08)	1024	3.05 (2.98–3.11)	1018	1.00	1.00	1.00
	Registered nurses	3.06 (2.97–3.15)	467	2.92 (2.86–2.98)	850	2.85 (2.80–2.90)	1520	0.011*	0.003*	0.434
	Nursing assistants	2.84 (2.68–3.01)	131	3.01 (2.93–3.09)	578	2.92 (2.86–2.97)	1256	0.163	1.00	0.206
Work–Life Interference^1^	Physicians	3.25 (3.14–3.36)	536	3.20 (3.13–3.26)	1022	3.15 (3.06–3.24)	1014	0.964	0.760	1.00
	Registered nurses	3.36 (3.24–3.47)	461	3.03 (2.95–3.10)	851	2.95 (2.88–3.01)	1513	0.001*	0.001*	0.570
	Nursing assistants	2.98 (2.78–3.18)	132	3.22 (3.13–3.32)	578	3.06 (2.99–3.12)	1241	0.068	1.00	0.027*
Job Resources										
Work content control^1^	Physicians	2.27 (2.14–2.40)	395	2.29 (2.21–2.36)	894	2.39 (2.30–2.49)	891	1.00	0.607	0.390
	Registered nurses	2.47 (2.29–2.65)	274	2.96 (2.85–3.07)	547	3.03 (2.94–3.12)	1035	0.001*	0.001*	1.00
	Nursing assistants	2.32 (2.03–2.60)	61	2.14 (2.02–2.27)	302	2.37 (2.27–2.47)	515	0.732	1.00	0.030*
Work time control^1^	Physicians	2.19 (2.06–2.32)	504	2.53 (2.45–2.61)	963	2.43 (2.33–2.54)	936	0.001*	0.056	0.630
	Registered nurses	2.00 (1.85–2.14)	440	2.43 (2.33–2.53)	781	2.37 (2.28–2.46)	1309	0.001*	0.001*	1.00
	Nursing assistants	2.13 (1.90–2.37)	111	2.06 (1.94–2.17)	482	2.03 (1.95–2.12)	940	1.00	1.00	1.00
Influence^1^	Physicians	3.50 (3.42–3.58)	518	3.58 (3.53–3.63)	985	3.69 (3.63–3.75)	973	0.189	0.008*	0.051
	Registered nurses	3.10 (3.01–3.19)	462	3.50 (3.44–3.56)	816	3.68 (3.62–3.73)	1436	0.001*	0.001*	0.001*
	Nursing assistants	2.85 (2.67–3.03)	107	2.90 (2.81–2.98)	513	3.09 (3.03–3.15)	1057	1.00	0.049*	0.002*
Social support—managers^1^	Physicians	3.71 (3.57–3.84)	496	3.64 (3.56–3.71)	995	3.61 (3.51–3.72)	963	0.919	1.00	1.00
	Registered nurses	3.58 (3.44–3.72)	457	3.61 (3.52–3.69)	835	3.53 (3.45–3.61)	1450	1.00	1.00	0.811
	Nursing assistants	3.53 (3.30–3.76)	128	3.49 (3.38–3.59)	569	3.54 (3.46–3.61)	1221	1.00	1.00	1.00
Social support—colleagues^1^	Physicians	4.35 (4.26–4.44)	537	4.33 (4.27–4.38)	1026	4.28 (4.20–4.35)	1002	1.00	0.854	0.983
	Registered nurses	4.37 (4.29–4.46)	465	4.43 (4.38–4.49)	851	4.46 (4.41–4.50)	1502	0.476	0.485	1.00
	Nursing assistants	4.30 (4.15–4.45)	132	4.23 (4.16–4.30)	583	4.29 (4.25–4.34)	1257	1.00	1.00	0.495

^*^Bonferroni *p* < 0.05

^†^Confounders include sex, birth country, working hours, place of work and county of work

Note that several study measurement scales were reverse-coded to ensure that higher scores uniformly indicate greater demands or greater resources. The original coding of all scales is documented in the supplementary material

Scales ranges in the table:

^1^Scale 1–5

^2^Scale 0–4

Registered nurses showed a clear pattern in which individuals with < 5 years of experience encountered more job demands and fewer resources than more experienced registered nurses. For example, compared with registered nurses > 15 years of experience who reported a WLI grand mean of 2.95 and an Influence grand mean of 3.68, registered nurses with < 5 years reported x̄ 3.36 and x̄ 3.10, respectively.

There were few significantly different patterns of experience with job demands and resources among assisted nurses with different years of working experience.

According to the stratification by employment sector (Table 4), compared with physicians working for a public employer, physicians with only significant differences in experience with Emotional Demands and those working for a private employer had greater Emotional Demands (x̄ 3.47) and less Collegial Support (x̄ 4.22).

**Table 4:** Mean values for job demands and job resources stratified working for a public or private employer for each profession adjusted by confounders†

		Public vs. private employment				
		Public		Private		Bonferroni p-value
		Mean (95% CI)	*n*	Mean (95% CI)	*n*	Public sector vs. private sector
Job Demands						
Quantitative Demands^1^	Physicians	3.25 (3.21–3.29)	2124	3.15 (3.06–3.25)	363	0.059
	Registered nurses	2.83 (2.79–2.86)	2400	2.80 (2.70–2.89)	307	0.545
	Nursing assistants	2.63 (2.59–2.67)	1756	2.83 (2.69–2.97)	172	0.009*
Emotional Demands^1^	Physicians	3.29 (3.24–3.34)	2123	3.47 (3.35–3.59)	367	0.006*
	Registered nurses	3.38 (3.33–3.42)	2407	3.42 (3.29–3.55)	309	0.541
	Nursing assistants	3.16 (311–3.22)	1764	3.25 (3.07–3.44)	171	0.371
Effort Reward Imbalance^2^	Physicians	1.25 (1.23–1.27)	2073	1.24 (1.19–1.29)	355	0.842
	Registered nurses	1.38 (1.36–1.41)	2359	1.24 (1.18–1.30)	300	0.001*
	Nursing assistants	1.44 (1.42–1.47)	1695	1.56 (1.45–1.67)	157	0.045*
Illegitimate Work Tasks—Unnecessary^1^	Physicians	3.21 (3.18–3.24)	2115	3.17 (3.09–3.25)	365	0.365
	Registered nurses	2.98 (2.95–3.02)	2385	2.81 (2.72–2.90)	305	0.001*
	Nursing assistants	2.94 (2.9–2.98)	1740	3.10 (2.97–3.24)	167	0.030*
Illegitimate Tasks—Unreasonable^1^	Physicians	3.05 (3.01–3.08)	2122	3.02 (2.94–3.10)	368	0.565
	Registered nurses	2.93 (2.89–2.96)	2397	2.72 (2.63–2.81)	307	0.001*
	Nursing assistants	2.92 (2.88–2.96)	1757	3.10 (2.96–3.25)	171	0.023*
Work–Life Interference^1^	Physicians	3.18 (3.14–3.23)	2115	3.25 (3.15–3.36)	367	0.258
	Registered nurses	3.06 (3.02–3.10)	2394	2.88 (2.76–2.99)	309	0.004*
	Nursing assistants	3.10 (3.05–3.15)	1747	3.15 (2.97–3.33)	167	0.606
Job Resources						
Work Content Control^1^	Physicians	2.33 (2.28–2.37)	1765	2.22 (2.11–2.33)	340	0.098
	Registered nurses	2.93 (2.87–2.99)	1513	3.02 (2.87–3.17)	256	0.244
	Nursing assistants	2.27 (2.20–2.35)	779	2.43 (2.18–2.67)	81	0.249
Work Time Control^1^	Physicians	2.40 (2.35–2.45)	1988	2.38 (2.26–2.51)	341	0.770
	Registered nurses	2.29 (2.24–2.34)	2142	2.56 (2.41–2.71)	280	0.001*
	Nursing assistants	2.05 (1.99–2.11)	1369	1.93 (1.71–2.15)	131	0.330
Influence^1^	Physicians	3.60 (3.57–3.63)	2025	3.61 (3.54–3.69)	364	0.724
	Registered nurses	3.51 (3.48–3.54)	2297	3.70 (3.61–3.79)	299	0.001*
	Nursing assistants	3.02 (2.98–3.06)	1506	3.01 (2.85–3.17)	141	0.895
Social support—managers^1^	Physicians	3.64 (3.59–3.69)	2040	3.66 (3.54–3.79)	354	0.705
	Registered nurses	3.53 (3.48–3.57)	2331	3.83 (3.70–3.97)	298	0.001*
	Nursing assistants	3.54 (3.48–3.60)	1712	3.31 (3.10–3.51)	172	0.031*
Social support—colleagues^1^	Physicians	4.33 (4.30–4.37)	2123	4.22 (4.14–4.31)	362	0.020*
	Registered nurses	4.43 (4.40–4.46)	2397	4.54 (4.46–4.62)	305	0.012*
	Nursing assistants	4.28 (4.24–4.32)	1764	4.26 (4.12–4.39)	174	0.765

^*^Bonferroni *p* < 0.05

^†^Confounders include sex, birth country, working hours, working experience, place of work and county of work

Note that several study measurement scales were reverse-coded to ensure that higher scores uniformly indicate greater demands or greater resources. The original coding of all scales is documented in the supplementary material

Scales ranges in the table:

^1^Scale 1–5

^2^Scale 0–4

Compared with registered nurses with a private employer, those working within the public employment sector reported higher means of ERI (x̄ 1.38), IWT (x̄ 2.98 & x̄ 2.93) and WLI (x̄ 3.06). Similarly, registered nurses with a public employer consequently also reported fewer resources than did those working within the private sector.

For nursing assistants, the pattern differed. Those working within the private employment sector reported higher grand means of job demands, including Quantitative Demands (x̄ 2.83), ERI (x̄ 1.56) and IWT (x̄ 3.10 & x̄ 3.10). The authors also reported a notably lower mean regarding Managerial Support (x̄ 3.31) than from nursing assistants working for a public employer.

## Discussion

In this study, we aimed to investigate variations in the psychosocial work environment among Swedish healthcare workers using the JD-R model. Overall, we found noticeable variations between and within the three groups of healthcare workers in Sweden, both concerning job demands and resources. Despite variations in education, responsibilities, and compensation between physicians, registered nurses, and nursing assistants, they often work side-by-side within the same employer context. While previous research has focused primarily on the implications of diverse demands and resources, this study lays a foundation for understanding the origins of work environment implications.

This study addresses an important gap in the literature, offering crucial insights into profession-specific health risks for Sweden’s three major healthcare professions. This allows for the potential development of tailored interventions aimed at safeguarding employee and patient well-being that account for this variation in the psychosocial working environment. Our results reveal the intricate relationship between experienced work environments, in the form of job demands and resources, and individual professional roles.

### Demands: variations among healthcare workers

Our study aligns with prior research revealing profession-specific demands. Physicians articulated pronounced Quantitative Demands [20, 27], and registered nurses experienced high levels of Emotional Demands [5, 10]. In contrast, nursing assistants reported a high imbalance between Efforts and Rewards and between WLI [25]. However, previous studies rarely compare within and between healthcare professions, which is why this study makes important contributions.

Our results diverge from those of Eriksson et al.’s study among Swedish nursing professions [26], which reported that registered nurses experience both greater demands and fewer resources than nursing assistants [26], contrary to our findings, in which nursing assistants recurrently reported a worse working environment than both physicians and registered nurses did. Methodological differences in sample size and time may contribute to these discrepancies. Our study used a larger and more representative sample of all registered nurses and nursing assistants working in Sweden, whereas Eriksson et al. [26] collected their sample from 2012 to 2014 with a total sample of 840 nurses. This emphasises the need for more comprehensive studies examining how each profession perceives its work environment in various contexts.

### Resources: nursing assistants at risk

Our findings reveal an apparent trend indicating that nursing assistants encounter a distinct shortage of resources. Particularly prominent was the pronounced lack of Control, notably over working hours, and Influence, differentiating them from their healthcare colleagues with longer educational backgrounds.

Physicians and registered nurses differ in their experience of resources; however, with their greater access to resources than nursing assistants, workers’ education level and status may be involved in explaining the differences between professions. In the general Swedish population, educational level has been shown to work as a predictor of experiencing Control, where more highly educated individuals tend to experience greater job control despite having more psychologically demanding jobs [52].

Moreover, given the established association between job resources and heightened work engagement and job satisfaction [5, 9, 31], our data prompt concerns about diminished work engagement among nursing assistants in contrast to their colleagues. This raises concerns about potential consequences, notably competence loss—a paramount issue concerning a profession already characterised by precarious employment and competence draught [53]. This is particularly noteworthy considering the integral role that nursing assistants play in the direct personal care of patients within the healthcare landscape.

### Re-evaluating the buffer hypothesis?

Our results emphasise the critical consideration of the balance between demands and resources across healthcare professions. While variations in demands among workers suggest the need to address profession-specific health risks, the more pronounced disparities in resource perception prompt a revisitation of previous research and the buffering hypothesis of the JD-R model.

The buffer hypothesis, integral to the JD-R model, posits that access to resources mitigates the adverse effects of confronting job demands [30]. In essence, individuals with elevated job demands risk exhaustion and those with limited job resources risk disengagement, while individuals in work roles with both high demands and limited resources face simultaneous exhaustion and disengagement [29]. However, as articulated by Marzocchi et al. [54], even in the face of moderate demands, access to resources significantly influences the impact of a demanding work environment on job satisfaction and well-being rather than the demands themselves [54]. This implies that although nursing assistants reported fewer Quantitative- and Emotional Demands than physicians and registered nurses did, their restricted resources may elevate their risk of experiencing both exhaustion and disengagement compared to their healthcare colleagues. This underscores the need for nuanced considerations beyond the linear buffer hypothesis.

Moreover, newly published studies have articulated the need to review and revise the buffer hypothesis [9, 32, 55]. Huth and Chung-Yan’s Bayesian meta-analysis in 2023 challenges the compensatory role of increased job control in high workload situations. Similar conclusions were established in a longitudinal study of Swedish workers [55], demonstrating that high demands correlate with an elevated risk of burnout irrespective of the level of the supportive environment (decision latitude and social capital). While our study did not directly test the demands–resources interaction, our results underscore the necessity for nuanced discussions, contextualising the work environment of each healthcare worker.

### Recognising additional contextual factors on demands and resources

Our results indicate significant variations in the psychosocial work environment among healthcare workers, both across different professions and within the same professional setting. Consistent with previous research [12, 36], we observed notable distinctions between private and public sector employment. Specifically, registered nurses employed in the public sector reported a less favourable work environment compared to their privately employed counterparts. However, public-sector nursing assistants reported experiencing lower job demands than those working in the private sector. Moreover, is that physicians in the private sector reported significantly higher Emotional Demands compared to those employed in the public sector. A majority of the physicians being privately employed work within primary care settings with financial incentives. Previous studies on healthcare provision in Sweden have shown that this leads to a perception of increased production of shorter visits and even skimming of patients [37], which could mean an increase in moral stress and explain the private physicians' prevalence in emotional demands. These results underscore the complexity of psychosocial work environments, suggesting that a uniform approach to addressing these challenges may not be appropriate. The observed differences not only vary by profession, but also within each profession based on employment context.

Additionally, our findings, consistent with prior research [56, 57], underscore a clear relationship between years of working experience and the psychosocial work environment. Fewer years of experience seem to be related to a poorer psychosocial work environment, which is particularly evident among registered nurses. Surprisingly, compared with their more experienced counterparts, physicians with less than 5 years of experience reported similar or even lower Quantitative- and Emotional Demands and fewer Unnecessary Tasks. This contrasts with earlier studies highlighting deteriorating conditions for junior physicians [1]. Explaining these nuanced differences, Dyrbye’s research suggested that, compared to early-career physicians (< 10 years), mid-career physicians (11–20 years) report a worsened psychosocial work environment. Educational factors may play a role, as fully qualified post-basic training nurses are immediately immersed in the demanding healthcare work environment, while physicians continue their education for several years post-basic training. This could afford physicians greater protection against certain job demands.

Once again, the divergences found in this study underscore the need to discard the uniform treatment of healthcare workers. Each profession and worker face unique stressors, influenced by contextual differences and individual compositions [9]. Our findings affirm that explicit demands and resources are shaped by both individual and profession-specific factors rooted in distinct job roles and experiences [8, 9, 33]. These insights emphasise the need for tailored interventions that address the unique challenges faced by each worker and profession, ultimately impacting the well-being of healthcare professions and, by extension, patient care.

### Strengths and limitations

This study has both strengths and limitations worth noting. First, the reliance on self-reported measures, with Emotional Demands and Social Support being measured by one item each, may introduce common method bias, potentially influencing the results. Second, the cross-sectional nature of the study limits our ability to investigate temporal changes in the psychosocial work environment between healthcare professions. On the other hand, the study aimed to uncover current differences, that is, to identify any possible pattern in the perception of the work environment. Additionally, the use of a representative and large analytical sample comprising 7589 individuals, including a division of three different professions physicians, registered nurses, and nursing assistants, is a major strength allowing for more generalisable and coherent results. Nonetheless, future research should adopt a longitudinal approach with multiple time intervals to assess the stability of these variations over time.

### Practical implications and future research

In practice, projections by Liu et al. indicate a critical global shortage of healthcare personnel, estimated at 80 million by 2030, partly attributed to adverse psychosocial work environments [58]. This shortage is evident in Nordic [18, 56] and Swedish healthcare settings [20, 26, 40, 59]. In addition to individual repercussions, these challenges compromise patient safety and quality care [11, 19, 22, 60, 61] and sustain significant societal and economic costs [62, 63]. Research [64] and legislation [65] emphasise the need for interventions to improve the well-being of employees, whereby the identification of relevant factors through risk assessments is imperative. Therefore, a comprehensive investigation of demands and resources and how they interact within explicit contextual settings across healthcare workers is imperative. Accounting for context, including how the individual, private life, job, and organisation interact in the experience of job demands and resources and their accumulative effect on health outcomes, could contribute to identifying profession-specific health risks and understanding their potential consequences for patient care.

## Conclusion

Our research emphasises the distinctive psychosocial work environments experienced by the three major healthcare professions in Sweden. We have shown that healthcare professionals, though often viewed as one group, experience vastly different psychosocial work environments, even those who often work under the same employer and the same unit. To safeguard both staff and patients, we must stop studying physicians, registered nurses, and nursing assistants in isolation. To identify profession-specific health risks and to understand their possible consequences for patient care, we need a unified approach, treating healthcare workers as different professions with different demands and resources but who are interdependent within the same healthcare context.

## Supplementary Information

Supplementary Material 1.

## Data Availability

The datasets analysed during the current study are not publicly available for ethical reasons but are available from the corresponding author upon reasonable request.

## Footnotes

1 The interactions of social structures, environmental exposures, and psychological processes within an individual’s work environment [21].

2 ER-ratio = effort score/(reward score x c); c = effort/reward = 3/7 = 0.42857143 [42].

3 A factor analysis was conducted to discern the relationship between 11 variables related to decision power over meetings with patients, over time sheets, length, start and end of shifts along with variables concerning time with patients and freedom for clinical decisions. The factor analysis produced 3 components (Eigenvalues > 1.0, …% of the variance). Concerning a factor loading over 0.3, components 1 and 2 were deemed as measuring two different forms of control: Work Content Control (component 1, 5 items) and Work time Control (component 2, 3 items). Component 3 was deemed as measuring feelings of influence at work (3 items).
