# Supplementary material for: Contextualising the job demands–resources model: a cross-sectional study of the psychosocial work environment across different healthcare professions
Source: Hum Resour Health. 2024 Nov 19;22:77. doi: 10.1186/s12960-024-00958-1 (PMC11577852; doi:10.1186/s12960-024-00958-1)
Supplement: Supplementary file 1 — Supplementary Material 1. [file 12960_2024_958_MOESM1_ESM.docx]

**SUPPLEMENTARY**

| **Supplementary Table A: Items, response alternatives and Cronbach's alpha for each study measure of job demand and job resource per healthcare profession** | | | | | | |
| --- | --- | --- | --- | --- | --- | --- |
| **Study Measurement** | | | **Cronbach’s Alpha** | | | |
| Measurement | Item | Response Alternatives | Total | Physician | Registered Nurses | Nursing assistants |
| Emotional Demands ^a^ | Do you have to deal with other people's (not colleagues') personal problems in your work? | 1=Always; 2=Often; 3=Sometimes; 4=Seldom; 5= Never/almost never | N/A | N/A | N/A | N/A |
| Quantitative Demands ^a^ | Is your workload unevenly distributed so that work piles up? | 1=Always; 2=Often; 3=Sometimes; 4=Seldom; 5= Never/almost never | .866 | .871 | .864 | .825 |
|  | How often do you not have time to complete all your tasks? | 1=Always; 2=Often; 3=Sometimes; 4=Seldom; 5= Never/almost never |  |  |  |  |
|  | Are you falling behind with your work? | 1=Always; 2=Often; 3=Sometimes; 4=Seldom; 5= Never/almost never |  |  |  |  |
| Effort Reward Imbalance ^a^ | I have constant time pressure due to a heavy workload. | 1=Strongly disagree; 2=Disagree; 3=Agree; 4=Strongly agree | .782 | .749 | .791 | .819 |
|  | I have many interruptions and disturbances while performing my job. | 1=Strongly disagree; 2=Disagree; 3=Agree; 4=Strongly agree |  |  |  |  |
|  | Over the past few years, my job has become more and more demanding. | 1=Strongly disagree; 2=Disagree; 3=Agree; 4=Strongly agree |  |  |  |  |
|  | I receive the respect I deserve from my superior or respective. | 1=Strongly disagree; 2=Disagree; 3=Agree; 4=Strongly agree | .769 | .781 | .752 | .714 |
|  | My job promotion prospects are poor † | 1=Strongly agree; 2=Agree; 3=Disagree; 4=Strongly disagree |  |  |  |  |
|  | I have experienced or expect to experience an undesirable change in my work situation † | 1=Strongly agree; 2=Agree; 3=Disagree; 4=Strongly disagree |  |  |  |  |
|  | My job security is poor † | 1=Strongly agree; 2=Agree; 3=Disagree; 4=Strongly disagree |  |  |  |  |
|  | Considering all my efforts and achievements, I receive the respect and prestige I deserve at work | 1=Strongly disagree; 2=Disagree; 3=Agree; 4=Strongly agree |  |  |  |  |
|  | Considering all my efforts and achievements, my job promotion prospects are adequate. | 1=Strongly disagree; 2=Disagree; 3=Agree; 4=Strongly agree |  |  |  |  |
|  | Considering all my efforts and achievements, my salary/income is adequate. | 1=Strongly disagree; 2=Disagree; 3=Agree; 4=Strongly agree |  |  |  |  |
| Illegitimate Work Tasks – Unreasonable ^a^ | How often do you have to perform tasks that you think ...should be done by someone else? | 1=Very often; 2=Often; 3=Sometimes; 4=Seldom; 5=Never | .770 | .764 | .767 | .781 |
|  | ...require more of you than is reasonable? | 1=Very often; 2=Often; 3=Sometimes; 4=Seldom; 5=Never |  |  |  |  |
|  | ...put you in unpleasant situations? | 1=Very often; 2=Often; 3=Sometimes; 4=Seldom; 5=Never |  |  |  |  |
|  | ...are unfairly assigned to you? | 1=Very often; 2=Often; 3=Sometimes; 4=Seldom; 5=Never |  |  |  |  |
| Illegitimate Work Tasks – Unnecessary ^a^ | How often do you have tasks that you wonder if they ...really need to be done at all? | 1=Very often; 2=Often; 3=Sometimes; 4=Seldom; 5=Never | .839 | .815 | .839 | .865 |
|  | ...sensible and meaningful? | 1=Very often; 2=Often; 3=Sometimes; 4=Seldom; 5=Never |  |  |  |  |
|  | ...should exist at all or could be done faster if things were organized differently? | 1=Very often; 2=Often; 3=Sometimes; 4=Seldom; 5=Never |  |  |  |  |
|  | ...should exist at all or could be done faster if some others made fewer mistakes? | 1=Very often; 2=Often; 3=Sometimes; 4=Seldom; 5=Never |  |  |  |  |
| Work-Life Interference | When I come home from work, I am too tired to do the things I would like to do. | 1=Not at all; 2=Rarely; 3=Sometimes; 4=Often; 5=Almost all the time | .927 | .943 | .928 | .921 |
|  | My private life does not look the way I would like it to because of my work. | 1=Not at all; 2=Rarely; 3=Sometimes; 4=Often; 5=Almost all the time |  |  |  |  |
|  | I overlook personal problems because of the demands of my work. | 1=Not at all; 2=Rarely; 3=Sometimes; 4=Often; 5=Almost all the time |  |  |  |  |
|  | My personal life suffers because of my work. | 1=Not at all; 2=Rarely; 3=Sometimes; 4=Often; 5=Almost all the time |  |  |  |  |
|  | I change and adapt my personal life to the demands of the workplace. | 1=Not at all; 2=Rarely; 3=Sometimes; 4=Often; 5=Almost all the time |  |  |  |  |
| Managerial Support ^a^ | If you need to, do you get support and help with your work from your line manager? | 1=Always; 2=Often; 3=Sometimes; 4=Rarely; 5=Never/almost never; 6=Not applicable | N/A | N/A | N/A | N/A |
| Collegial Support ^a^ | If you need to, do you get help and support from your colleagues? | 1=Always; 2=Often; 3=Sometimes; 4=Rarely; 5=Never/almost never; 6=Not applicable | N/A | N/A | N/A | N/A |
| Influence ^a^ | In my workplace, I have...enough time with my patients during a typical patient encounter. | 1=To a very high degree; 2=To a fairly high degree; 3=To neither a high nor a low degree; 4=To a fairly low degree; 5=To a very low degree; 6=Not applicable | .777 | .706 | .788 | .800 |
|  | ... freedom to make clinical decisions that meet the patient's needs. | 1=To a very high degree; 2=To a fairly high degree; 3=To neither a high nor a low degree; 4=To a fairly low degree; 5=To a very low degree; 6=Not applicable |  |  |  |  |
|  | ... the opportunity to provide high-quality care to all patients. | 1=To a very high degree; 2=To a fairly high degree; 3=To neither a high nor a low degree; 4=To a fairly low degree; 5=To a very low degree; 6=Not applicable |  |  |  |  |
| Work Time Control ^a^ | How much can you influence your working hours based on ... the length of your shifts? | 1=To a very high degree; 2=To a fairly high degree; 3=To neither a high nor a low degree; 4=To a fairly low degree; 5=To a very low degree; 6=Not applicable | .911 | .907 | .910 | .917 |
|  | ... the start of your shifts? | 1=To a very high degree; 2=To a fairly high degree; 3=To neither a high nor a low degree; 4=To a fairly low degree; 5=To a very low degree; 6=Not applicable |  |  |  |  |
|  | ... the end of your shifts? | 1=To a very high degree; 2=To a fairly high degree; 3=To neither a high nor a low degree; 4=To a fairly low degree; 5=To a very low degree; 6=Not applicable |  |  |  |  |
| Work Content Control ^a^ | To what extent do you have control over… which patients you see? | 1=To a very high degree; 2=To a fairly high degree; 3=To neither a high nor a low degree; 4=To a fairly low degree; 5=To a very low degree; 6=Not applicable | .909 | .906 | .910 | .917 |
|  | ... how many patients you see during the day? | 1=To a very high degree; 2=To a fairly high degree; 3=To neither a high nor a low degree; 4=To a fairly low degree; 5=To a very low degree; 6=Not applicable |  |  |  |  |
|  | ... the amount of time for patient appointments? | 1=To a very high degree; 2=To a fairly high degree; 3=To neither a high nor a low degree; 4=To a fairly low degree; 5=To a very low degree; 6=Not applicable |  |  |  |  |
|  | ... time for administration or documentation? | 1=To a very high degree; 2=To a fairly high degree; 3=To neither a high nor a low degree; 4=To a fairly low degree; 5=To a very low degree; 6=Not applicable |  |  |  |  |
| a. Study measurement scales that were reverse-coded for the analysis to ensure that higher scores uniformly indicate greater demands or greater resources. | | | | | | |
| † Reverse-coded items in analysis to create the Effort Reward Imbalance Scale ratio | | | | | | |
